# The adaptive evolution of the mammalian mitochondrial genome

**DOI:** 10.1186/1471-2164-9-119

**Published:** 2008-03-04

**Authors:** Rute R da Fonseca, Warren E Johnson, Stephen J O'Brien, Maria João Ramos, Agostinho Antunes

**Affiliations:** 1 REQUIMTE, Departamento de Química, Faculdade de Ciências, Universidade do Porto, Rua do Campo Alegre, 687, 4169-007 Porto, Portugal; 2 Evolutionary Biology, Institute of Biology, University of Copenhagen, Universitetsparken 15, 2100 Copenhagen Ø, Denmark; 3 Laboratory of Genomic Diversity, National Cancer Institute, Frederick, MD 21702-1201, USA; 4 CIMAR/CIIMAR, Centro Interdisciplinar de Investigação Marinha e Ambiental, Universidade do Porto, Rua dos Bragas, 177, 4050-123 Porto, Portugal

## Abstract

**Background:**

The mitochondria produce up to 95% of a eukaryotic cell's energy through oxidative phosphorylation. The proteins involved in this vital process are under high functional constraints. However, metabolic requirements vary across species, potentially modifying selective pressures. We evaluate the adaptive evolution of 12 protein-coding mitochondrial genes in 41 placental mammalian species by assessing amino acid sequence variation and exploring the functional implications of observed variation in secondary and tertiary protein structures.

**Results:**

Wide variation in the properties of amino acids were observed at functionally important regions of cytochrome *b *in species with more-specialized metabolic requirements (such as adaptation to low energy diet *or *large body size, such as in elephant, dugong, sloth, and pangolin, and adaptation to unusual oxygen requirements, for example diving in cetaceans, flying in bats, and living at high altitudes in alpacas). Signatures of adaptive variation in the NADH dehydrogenase complex were restricted to the loop regions of the transmembrane units which likely function as protons pumps. Evidence of adaptive variation in the cytochrome *c *oxidase complex was observed mostly at the interface between the mitochondrial and nuclear-encoded subunits, perhaps evidence of co-evolution. The ATP8 subunit, which has an important role in the assembly of F_0_, exhibited the highest signal of adaptive variation. ATP6, which has an essential role in rotor performance, showed a high adaptive variation in predicted loop areas.

**Conclusion:**

Our study provides insight into the adaptive evolution of the mtDNA genome in mammals and its implications for the molecular mechanism of oxidative phosphorylation. We present a framework for future experimental characterization of the impact of specific mutations in the function, physiology, and interactions of the mtDNA encoded proteins involved in oxidative phosphorylation.

## Background

Mitochondrial DNA (mtDNA) has long been treated as an ideal marker because of its convenience for reconstruction of gene genealogy and population history inference. However, the selective neutrality assumption of the mtDNA is simplistic because variation in mitochondrial protein-coding genes involved in oxidative phosphorylation (responsible for the production of up to 95% of the energy of eukaryotic cells) can directly influence metabolic performance. Because of the importance of this biochemical pathway, evaluating selective pressures acting on mtDNA proteins could provide key insight into the adaptive evolution of the mtDNA genome as has been suggested by recent empirical evidence (e.g. Ruiz-Pesini et al. 2004 [1], Moyer et al. 2005 [2], Bazin et al. 2006 [3]). As amino acid changes cause inefficiencies in the electron transfer chain system, oxidative phosphorylation produces reactive oxygen molecules, causing oxidative damage to mitochondrial and cellular proteins, lipids and nucleic acids, and eventually interrupting the production of mitochondrial energy.

Mutations in mitochondrial-encoded genes can influence the production of reactive oxygen species in mice [4] and have been implicated in a large number of human and mouse diseases [5]. Some amino acid changes may also improve aerobic capacity and adaptation to new thermal environments [6-11]. Furthermore, mutations in mitochondrial genes have been implicated in exercise intolerance in humans [12] (see Table 1). Metabolic capacity varies widely among mammalian species [13], and variation in the elements of the oxidative phosphorylation pathway have been linked to different life history traits and environmental adaptations [14-16]. In addition, functional interactions between mitochondrial- and nuclear-encoded proteins may result in co-adaptation to maintain or improve metabolic fitness [9,17].

**Table 1:** Examples of mutations in mitochondrial genes that cause exercise intolerance in humans.

Gene	Mutation	Codon	Comments	References
**ND1**	**Ala→Thr**	**52**		[75]

	**Thr→Ala**	**164**		[76]

ND4	Trp→TER	358		[77]

**COX1**	**Trp→TER**	**6**		[78]

COX2	Met→Lys	29	First N-terminal membrane-spanning region of COXII: a structural association of COXII with COXI is necessary to stabilize the binding of heme a3 to COXI.	[79]

**COX3**	**Trp→TER**	**249**	**Loss of the last 13 amino acids of the highly conserved C-terminal region of this subunit.**	[80]

	**Trp→TER**	**58**		[81]

CYTB	Gly→Asp	290	Q_o _binding pocket	[82]

	Gly→Ser	34	Heme b_h _and Q_i _binding pocket	[83]

	Trp→TER	135		[84]

	Ser→Pro	151	Disrupts helix cd_1_, Qo site	[84]

	Gly→Asp	251	Heme b_l_	[85]

	Ser→Pro	35	Disrupts helix A, Q_i _pockect	[86]

**Figure 1:**
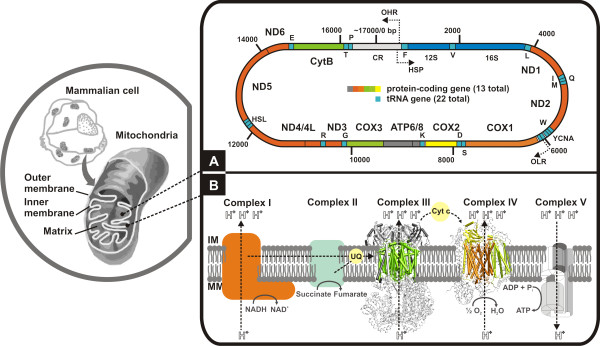
**The mammalian mitochondrial genome and its protein-coding gene repertoire involved in the oxidative phosphorylation pathway**. **(A) **Schematic representation of genes within mammalian mitochondrial genome (~7,000 bp). Genes on the outer circle are transcribed from the light-strand. Location of the tRNAs (red boxes) conform to the canonical placental mammalian arrangement. Abbreviations: HSP, putative heavy-strand promoter; OHR, origin of heavy-strand replication; OLR, origin of light-strand replication. **(B) **Simplified view of the mitochondrial oxidative phosphorylation machinery. Complexes I (NADH dehydrogenase) and II (succinate dehydrogenase) receive electrons from either NADH or FADH_2_. Electrons are then carried between complexes by the carrier molecules coenzyme Q/ubiquinone (UQ) and cytochrome c (CYC). The potential energy of these electron transfer events is used to pump protons against the gradient, from the mitochondrial matrix into the intermembrane space [Complexes I and III (cytochrome *bc*_1_) and IV (cytochrome *c *oxidase)]. ATP synthesis by Complex V (ATP synthase) is driven by the proton gradient, and occurs in the mitochondrial matrix. MM: mitochondria matrix; IM: intermembrane space.

The mtDNA comprises a closed circular DNA strand that encodes the following proteins involved in the oxidative phosphorylation machinery: seven subunits of the NADH dehydrogenase or NADH ubiquinone oxidoreductase complex (ND: ND1, 2, 3, 4, 4L, 5 and 6), the cytochrome *b *subunit of the ubiquinol cytochrome c oxidoreductase or cytochrome *bc*_1 _complex (CytB), three subunits of the cytochrome *c *oxidase complex (COX), and two subunits of ATP synthase (ATPase: ATP6 and ATP8) [18] (Figure 1A). These key components of four of the five complexes involved in oxidative phosphorylation, combine with other subunits encoded by the nuclear DNA genome [18] (Figure 1B). In the oxidative phosphorylation, reducing equivalents resulting from the oxidation of nutrients such as glucose, are carried by a series of molecules (the electron transport chain), which have increasing standard reduction potentials. The resulting free energy is transformed into a proton gradient that is used by ATP synthase to produce ATP (adenosine triphosphate) from ADP (adenosine diphosphate) [18].

Although mtDNA sequence data have been extensively used in mammalian phylogenetics [19-24], relatively little attention has been devoted to the study of molecular adaptation of mitochondrial encoded proteins. In this work we combine molecular evolution analyses with crystallographic and secondary structure prediction analyses to explore how mitochondrial genetic variation may be linked to the diverse metabolic patterns of 41 mammalian species (Table 2) from each of the four major clades of the placental mammals. Since most of the cell's ATP production results from mitochondrial oxidative phosphorylation, we also use estimates of metabolic rates based on oxygen (O_2_) consumption under aerobic conditions as a surrogate for physiological or metabolic differences among the species in our analyses.

**Table 2:** The 41 mammalian species in study.

Common name	Acession number	Superorder (Order)	Species	Habitat	Food Habits	Geographic Range	Basal Metabolic Rate [53, 87] (ml of O2 per h); M (g)
Cape Golden Mole	NC_004920	Afrotheria (Chrysochloridae)	*Chrysochloris asiatica*	sandy soil	small invertebrates	South Africa	low; 44

**cape rock hyrax**	**NC_004919**	**Afrotheria (Hyracoidea)**	***Procavia capensis***	**savanna or grassland; scrub forest.**	**herbivore**	**Syria south through NE Africa through most of sub-Saharan Africa. Isolated mountains in Libya and Algeria.**	**medium; 2400**

African savana elephant	NC_000934	Afrotheria (Proboscidea)	*Loxodonta africana*	savannah/desert	herbivore	Sahara Desert to the south tip of Africa, from the Atlantic (western) coast of Africa to the Indian Ocean in the east	high; 3670000 (from Elephas maximus)

**elephant shrew**	**NC_004921**	**Afrotheria (Macroscelidea)**	***Elephantulus sp. VB001***	**savannas, deserts, thornbush and tropical forests**	**inverterbrates**	**Africa**	**low; 51 (*Elephantus *AVE)**

short-eared elephant shrew	NC_004026	Afrotheria (Macroscelidea)	*Macroscelides proboscideus*	desert and semi-desert areas	invertebrates and herbivorous diet	Namibia, southern Botswana, and South Africa	low; 39

**Dugong**	**NC_003314**	**Afrotheria (Sirenia)**	***Dugong dugon***	**tropical marine coastal water**	**sea grasses**	**east Africa, northern coast of Australia, island groups of the South Pacific**	**UNK; 569000**

small Madagascar hedgehog	NC_002631	Afrotheria (Tenrecidae)	*Echinops telfairi*	dry forests, scrub, cultivated areas, dry coastal regions and semidesert	invertebrates; also baby mice	Madagascar	medium; 116

**Aardvark**	**NC_002078**	**Afrotheria (Tubulidentata)**	***Orycteropus afer***	**dry savanna to rain forest**	**omnivorous; termites**	**Africa**	**medium; 48000**

Malayan flying lemur	NC_004031	Euarchontoglires (Dermoptera)	*Cynocephalus variegatus*	arboreal	herbivorous	Thailand and Indochina	UNK; 1375

**Rabbit**	**NC_001913**	**Euarchontoglires (Lagomorpha)**	***Oryctolagus cuniculus***	**savanna or grassland; forest**	**herbivore**	**Europe**	**medium; 2000**

American pika	NC_005358	Euarchontoglires (Lagomorpha)	*Ochotona princeps*	areas of broken rock and talus; taiga; mountains	herbivore	central British Columbia to south-central California and east to Colorado	medium; 109

**domestic guinea pig**	**NC_000884**	**Euarchontoglires (Rodentia)**	***Cavia porcellus***	**grassy plains**	**herbivore**		**medium; 629**

greater cane rat	NC_002658	Euarchontoglires (Rodentia)	*Thryonomys swinderianus*	along river banks and near marshes	herbivore	KwaZulu-Natal; Gauteng and the Northern Province; Mpumalanga	UNK; 8

**house mouse**	**NC_005089**	**Euarchontoglires (Rodentia)**	***Mus musculus***	**temperate; forest**	**omnivore**	**originally distributed from the Mediterranean region to China; spread throughout the world**	**low; 21**

Rat	NC_001665	Euarchontoglires (Rodentia)	*Rattus norvegicus*	temperate; tropical; desert; savanna or grassland; chaparral; forests; mountains	omnivore	native to northern China; can be found on every continent of the world except Antarctica	medium; 300

**Eurasian red squirrel**	**NC_002369**	**Euarchontoglires (Rodentia)**	***Sciurus vulgaris***	**temperate; forest**	**herbivore**	**Europe and northern Asia**	**medium; 532 (*Sciurus *AVE)**

Human	NC_001807	Euarchontoglires (Primates)	*Homo sapiens*		omnivore		high; 75

**ring-tailed lemur**	**NC_004025**	**Euarchontoglires (Primates)**	***Lemur catta***	**tropical forests**	**herbivore**	**Madagascar**	**UNK**

northern tree shrew	NC_002521	Euarchontoglires (Scandentia)	*Tupaia belangeri*	tropical forests	omnivore	southeast Asia	medium; 123 (from *T. glis*)

**Dog**	**NC_002008**	**Laurasiatheria (Carnivora)**	***Canis familiaris***		**carnivore**		**medium; 8860 (*Canis *AVE)**

Cat	NC_001700	Laurasiatheria (Carnivora)	*Felis catus*		carnivore		high; 13200 (*Felidae *AVE)

**humpback whale**	**NC_006927**	**Laurasiatheria (Cetartiodactyla)**	***Megaptera novaeangliae***	**warm tropical waters to arctic waters**	**carnivores (crustaceans, plankton, and small fish)**	**North Pacific Ocean, North Atlantic Ocean, southern Hemisphere**	**UNK; 36000000**

white-beaked dolphin	NC_005278	Laurasiatheria (Cetartiodactyla)	*Lagenorhynchus albirostris*		carnivore (fish, squid, octopus and small crustaceans)	Icelandic waters and in the North Sea.	UNK; 200

**Hippopotamus**	**NC_000889**	**Laurasiatheria (Cetartiodactyla)**	***Hippopotamus amphibius***	**shallow water; can live in cold climates (but not frozen water)**	**herbivore**	**Africa**	**UNK; 235E+04**

Cattle	NC_006853	Laurasiatheria (Cetartiodactyla)	*Bos taurus*		herbivore		high; 500

**Pig**	**NC_000845**	**Laurasiatheria (Cetartiodactyla)**	***Sus scrofa***		**omnivore**		**high; 200**

Alpaca	NC_002504	Laurasiatheria (Cetartiodactyla)	*Lama pacos*	altitude of 3500 to 5000 meters above sea-level	herbivore	Andes	UNK; 170

**Ryukyu flying fox**	**NC_002612**	**Laurasiatheria (Chiroptera)**	***Pteropus dasymallus***		**fruit**		**medium; 492 (*Pteropus *AVE)**

Egyptian rousette	NC_007393	Laurasiatheria (Chiroptera)	*Rousettus aegyptiacus*	humid dark roosts	very ripe fruit	Africa, Egypt to Turkey, Cyprus, Arabian peninsula east to Pakistan	medium; 146

**Jamaican fruit-eating bat**	**NC_002009**	**Laurasiatheria (Chiroptera)**	***Artibeus jamaicensis***	**neotropical; forests**	**herbivore; also insects**	**central Mexico to Bolivia and central Brazil through the Greater and Lesser Antilles**	**low; 45**

New Zealand long-tailed bat	NC_002626	Laurasiatheria (Chiroptera)	*Chalinolobus tuberculatus*		insectivore	New Zealand	Low; 18 (from *C. gouldii*)

**western European hedgehog**	**NC_002080**	**Laurasiatheria (Eulipotyphla)**	***Erinaceus europaeus***	**savanna or grassland; forest**	**omnivore**	**region, except the Himalayas and North Africa**	**medium; 750**

long-clawed Shrew	NC_005435	Laurasiatheria (Eulipotyphla)	*Sorex unguiculatus*	wet grasslands to montane forests	seeds, insects, nuts, worms	along the Pacific coastline of Siberia	low; 13

**European mole**	**NC_002391**	**Laurasiatheria (Eulipotyphla)**	***Talpa europaea***	**savanna or grassland; forest**	**invertebrates**	**throughout temperate Europe to east in Russia**	**medium; 100**

Horse	NC_001640	Laurasiatheria (Perissodactyla)	*Equus caballus*		herbivore		high; 500

**white rhinoceros**	**NC_001808**	**Laurasiatheria (Perissodactyla)**	***Ceratotherium simum***	**savanna or grassland; chaparral ; forest**	**herbivore**	**Africa**	**UNK; 2700**

Brazilian tapir	NC_005130	Laurasiatheria (Perissodactyla)	*Tapirus terrestris*	forests	bulk of their diet is herbivorous; aquatic organisms	South America	UNK; 2000000

**long-tailed pangolin**	**NC_004027**	**Laurasiatheria (Pholidota)**	***Manis tetradactyla***	**rainforest**	**invertebrates**	**Uganda to Senegal and Angola**	**medium; 1430**

nine-banded armadillo	NC_001821	Laurasiatheria (Xenarthra)	*Dasypus novemcinctus*	savanna or grassland; forest	invertebrates; occasionally birds, small mammals, fruits	Peru and northern Argentina to the south-central and southeastern United States. It is also found on the islands of Grenada, Trinidad and Tobago	medium; 3510

**southern two-toed sloth**	**NC_006924**	**Laurasiatheria (Xenarthra)**	***Choloepus didactylus***	**rainforest**	**herbivore**	**Central America and northern South America**	**medium; 3770 (from *C. hoffmanni*)**

southern tamandua	NC_004032	Laurasiatheria (Xenarthra)	*Tamandua tetradactyla*	savanna or grassland; forests; at elevations to 2000 m	invertebrates	South America	medium; 3500

*AVE*	*average*						

*UNK*	*missing data*						

## Results and Discussion

**Figure 2:**
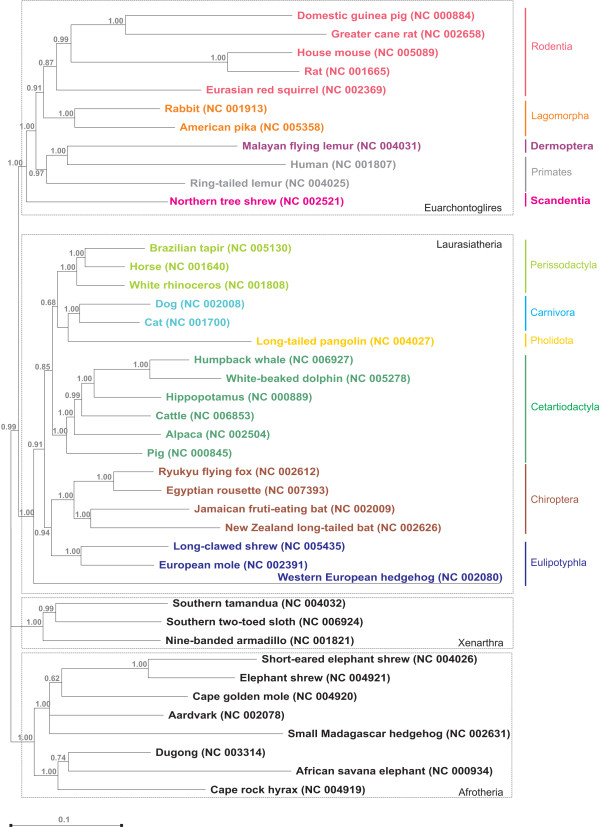
**Mammalian mitogenomic phylogenetic tree**. Consensus phylogenetic tree (50 percent majority rule) constructed from the combined set of MCMC runs resulting topologies in MrBayes. Numbers give the percentage of posterior probability support for each clade averaged over the runs.

The mitogenomic phylogenetic reconstruction (Figure 2) obtained with Bayesian inference methods [25,26] is mostly congruent with previous comprehensive analyses from nuclear and mtDNA genes [27] where mammalian species form four major groups: Laurasiatheria, Euarchontoglires, Xenarthra, and Afrotheria. The superordinal tree is resolved with posterior probabilities greater than 0.99. The relative positioning of the orders is in agreement with that obtained using Maximum Likelihood [see Additional file 1, Fig. S2], supporting the previous topology.

**Figure 3:**
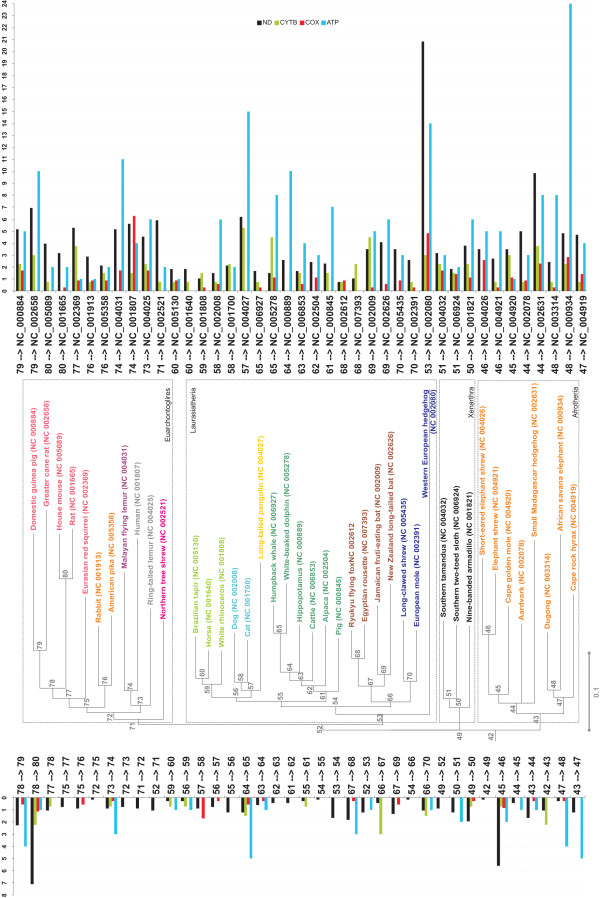
**Radical physicochemical amino acid changes varying across the mammalian mitogenomic phylogenetic tree**. Representation of the standardized number of strong positively selected amino acid properties across mitochondrial protein-coding genes varying within the branches of the mammalian mitochondrial tree (see Methods for details).

**Figure 4:**
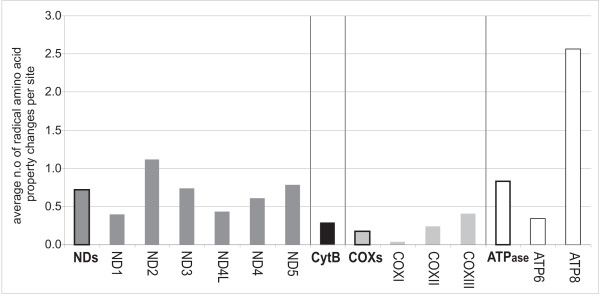
**Radical physicochemical amino acid changes among residues in mammalian mitochondrial protein-coding genes**. Number of strong positively selected amino acid properties in the mammalian mtDNA protein-coding genes of the oxidative phosphorylation chain.

Significant physicochemical amino acid changes among residues in mitochondrial protein coding genes were identified by the algorithm implemented in TreeSAAP [28], which compares the observed distribution of physicochemical changes inferred from a phylogenetic tree with an expected distribution based on the assumption of completely random amino acid replacement expected under the condition of selective neutrality (see details in the Methods section). There are more modifications in the number of radical amino acid property changes in the tips (80%) relative to the interior branches (20%) of the mammalian tree (Figure 3). The interior branches with the highest number of radical amino acid property changes are those that lead to the house mouse and the rat (node 78 to 80; a total of 55 changes across all proteins), the branch leading to the elephant shrews (node 45 to 46; 43 changes across all proteins), the branch leading to the guinea pig and the greater cane rat (node 78 to 79; 21 changes across all proteins), and the branch uniting the humpback whale and white-beaked dolphin (node 64 to 65; 17 changes across all proteins). There was an average of 10 changes per interior branch. The proteins with the highest average number of changing properties per site are ND and ATPase (0.7 and 0.8 average changes per site, respectively), while CytB and COX were the lowest (0.3 and 0.2 changes per site, respectively) (Figure 4). Noteworthy, a distinction arises between loop areas and transmembrane domains for all proteins complexes. Loop areas are, in general, more affected by positive selection than transmembrane domains (0.52 property changes showing strong positive selection per site *vs *0.39 for the latter).

**Figure 5:**
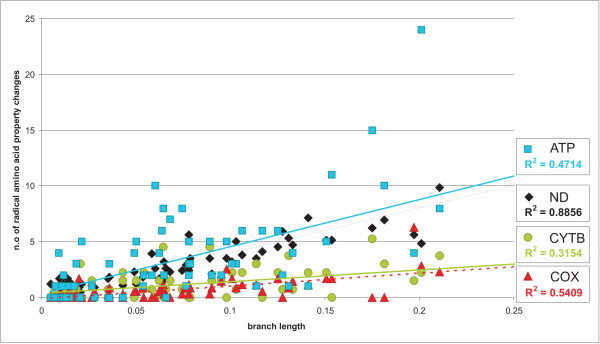
**Correlation between amino acid property variation and genetic distance**. Correlation between the number of positively selected amino acid properties and the branch length (genetic distance) in mammalian NDs, CytB, COXs, and ATPs protein-coding genes.

Amino acid properties with signals of strong positive selection accumulated at a rate roughly equivalent to the mutation rate of the gene itself (i.e. mutation rate of ATP > ND > CytB > COX; see Lopez et al. 1997 [29]) (Figure 5; [see Additional file 1, Fig. S3]). This correlation is more pronounced for the protein-coding genes with higher mutation rates, such as ATPase and ND, as is most apparent in analyses of the variation along the interior branches, where 20% of the radical amino acid property changes occur [see Additional file 1, Fig. S3]. The best correlation between overall mutation rate and number of sites with radical amino acid changes was observed for NDs, while the existence of several outliers for ATPase slightly reduced the strength of the correlation [see Additional file 1, Fig. S3].

**Figure 6:**
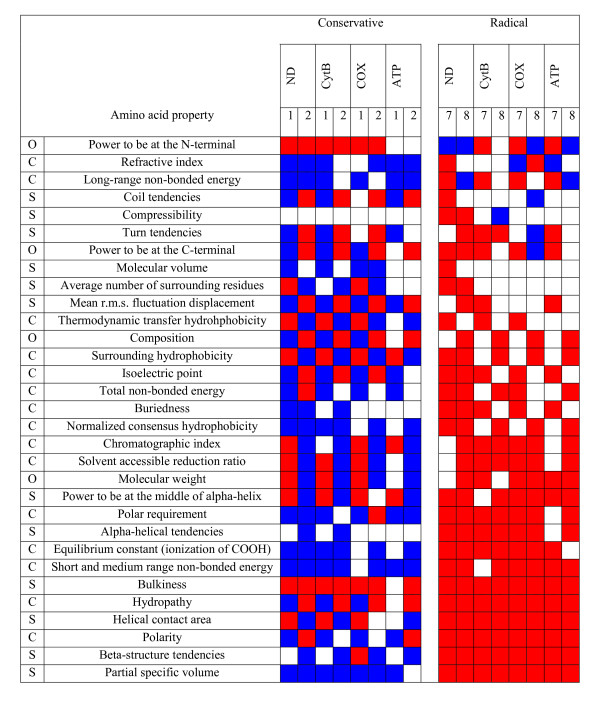
**Amino acid properties under positive (blue) and negative (red) selection in mammalian mitochondrial protein-coding genes**. Conservative changes correspond to conservative categories 1 and 2 and radical changes to categories 7 and 8 (*P *≤ 0.001). (C: chemical; S: structural; O: other [1])

**Figure 7:**
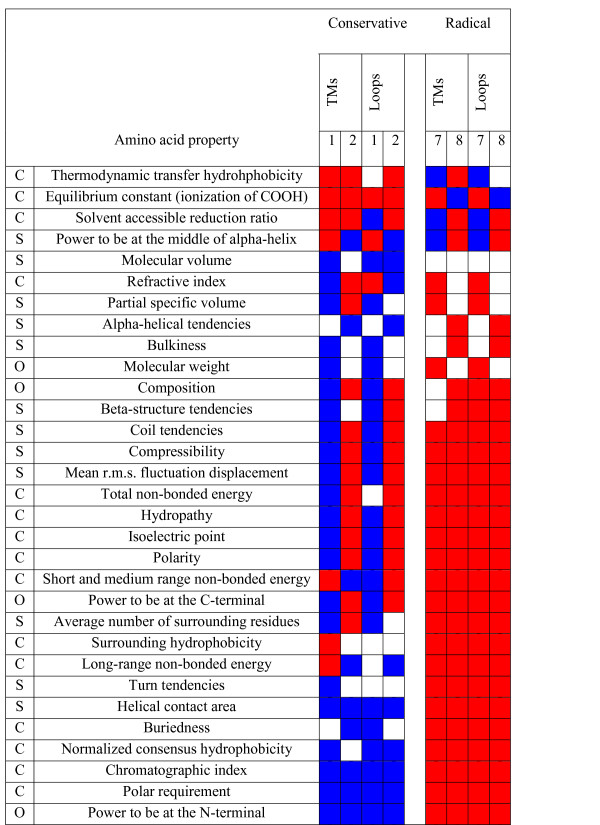
**Amino acid properties under positive (blue) and negative (red) selection in transmembrane and loop regions**. Conservative changes correspond to conservative categories 1 and 2 and radical changes to categories 7 and 8 (*P* ≤ 0.001). (C: chemical; S: structural; O: other [1])

The biochemical complexity of the oxidative phosphorylation processes precludes a clear discussion on the functional implications of the amino acid properties that are under selection (Figures 6 and 7). Negative selection dominates the categories of radical changes. Moderate changes (categories 1 and 2) characterize most of the positive selection detected. Properties under positive destabilizing selection (the power to be at the N-terminal, refractive index, long-range non-bonded energy, coil tendencies, compressibility, turn tendencies and the power to be at the C-terminal) will interfere both at a chemical and structural level. However, since we are considering events as diverse as protein-protein interactions, molecular oxygen and proton diffusion and electron-transfer, it is not possible to establish a direct correlation with the previously referred properties. Discussion on varying amino acid properties will therefore be made specifically for those sites which can be mapped on available structural data.

### NADH dehydrogenase

**Figure 8:**
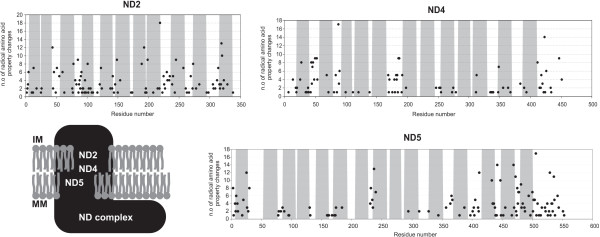
**Amino acid property variation in NADH dehydrogenase subunits ND2/3/5**. Topological assignment of the sites that present a high number of radically changing properties under positive-destabilizing selection in three subunits of the NADH dehydrogenase complex that are suggested to be proton pumping devices. The transmembrane domain average prediction is shown in grey (for details see Material and Methods section). MM: mitochondria matrix; IM: intermembrane space.

The sites with the highest number (between 17 and 18) of strong positively selected changes in amino acid properties are within ND2, ND4 and ND5 [see Additional file 1, Fig. S4]. These subunits show a high average in the number of such changes per residue (1.1, 0.6 and 0.8, respectively, for an overall average of 0.5) (Figure 4). NADH dehydrogenase is the first (Figure 1B) and the largest enzyme complex in the respiratory chain. It receives electrons from the oxidation of NADH and provides electrons for reduction of quinone to quinol. This is coupled to the translocation of four protons across the inner membrane, generating an electrochemical proton gradient (Figure 1B). Complex I is an L-shaped complex (as shown in low resolution electron microscopy analysis [30]) that contains all seven mtDNA encoded subunits in a membrane-embedded arm. The subunits of the "peripheral arm" (that projects into the mitochondrial matrix) are encoded by the nuclear DNA genome. Forty-six different units have been identified in complex I from bovine heart mitochondria [31], although the 14 bacterial subunits are the minimum needed for sufficient energy transduction by complex I [32]. The electron transfer chain events occur in the peripheral arm subunits and the proton pumping occurs in the membrane-embedded module. ND1 and ND2 have been suggested to be located at the junction between the peripheral and the membrane arm [32], while ND4 and ND5 should occur at the distal end of the latter [31]. ND2, ND4, and ND5 are suggested to be the actual proton pumping devices because of their sequence homology with a class of Na^+^/H^+ ^antiporters [32]. The overall variation in these subunits, which have already been assigned some function, is larger than the observed variation in the subunits with still unknown functions. Mutations in these subunits may interfere with the efficiency of the proton-pumping process. This could either occur through chemical changes that hinder/improve the proton translocation or by disrupting/improving the long-range redox-linked conformational changes that are suggested to occur in order to, for example, activate ND5 which is far away from the electron transfer events [32]. The number of TM domains predicted in this study (Figure 8) for ND2, ND4, and ND5 is similar to that present in their bacterial counterparts (which are 11, 12, and 17, respectively) [33]. Figure 8 shows that the sites with higher variation (20 < number of changes < 110) are located only in the loop regions, suggesting that functional constraints are acting upon the TM domains, which would be consistent with their putative proton-pumping function.

### Cytochrome *b*

**Figure 9:**
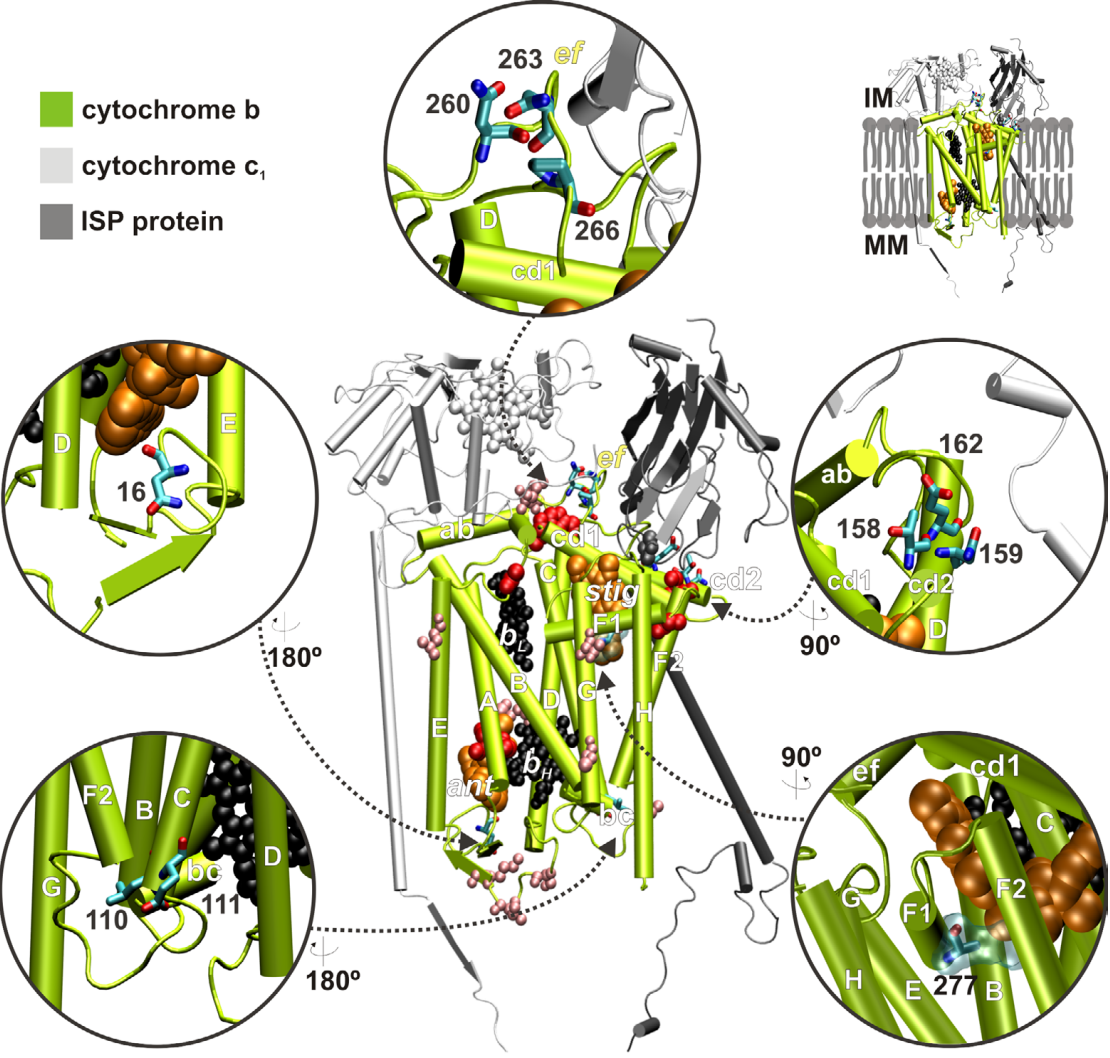
**Three-dimensional representation of relevant variable amino acids in mammalian cytochrome *b***. Illustrative representation of some of the amino acid variable sites (see **Figure 10**) located in relevant functional spots of the bovine CytB structure (pdb code: 1PPJ [66]). The prosthetic groups in CytB are represented in black, and the Q_i _and Q_P _bound inhibitors in orange (ant: antimycin; stig: stigmatellin). Mutations in sites shown in red have been related to exercise intolerance in humans (see Table 1). Mutations in sites shown in pink are under strong positive selection according to the TreeSAAP analysis (over 5 positively selected properties). In site 277, the alanine present in the bovine structure is shown as ball and stick and the van der Waals surface for the arginine found in dugong is also depicted. MM: mitochondria matrix; IM: intermembrane space.

CytB is an extremely conserved protein, reflecting its fundamental role in energy production in the mitochondria. It catalyses reversible electron transfer from ubiquinol to cytochrome *c *coupled to proton translocation (Q-cycle [34,35]). A quinol molecule at the Q_0 _site donates an electron to cytochrome *c *via the iron-sulfur protein (ISP) and cytochrome *c*_1 _(Figure 9). A second electron passes sequentially through the b_L _and b_H _heme ending up in a quinone/semiquinone radical at the Q_i _site. In a complete Q cycle, two quinol molecules are the Q_0 _site and one molecule of quinol is regenerated while four protons are translocated across the membrane. Available x-ray data showing cytochrome *bc*_1 _inhibitors bound to both Q_i _and Q_0 _sites suggest that these mutations have functional consequences. The high degree of conservation among CytB sequences (Figure 4) made it difficult to find evidence of positive selection using TreeSAAP. Sites detected by TreeSAAP were mainly located at the interface between mitochondrial and nuclear-encoded subunits (Figure 9). However, insight into the evolution of this protein was obtained by inspection of the amino acid substitutions observed in the sequence alignment of the various mammalian species.

**Figure 10:**
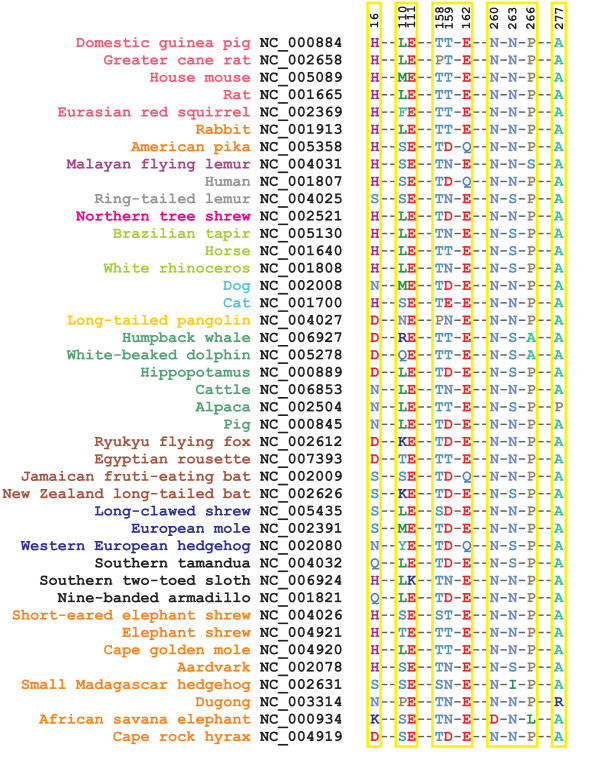
**Amino acid variation in functional sites of mammalian cytochrome *b***. Amino acid variation in the selected sites of CytB presented in **Figure 9 **across all the mammalian species surveyed in this study.

The African savanna elephant *Loxodonta Africana *has two atypical amino acid replacements at positions 16 and 260 (Figure 10) that are conserved in other members of the *Elephantidae *family (the African forest elephant *Loxodonta cyclotis*, the Asian elephant *Elephas maximus*, and both the extinct woolly mammoth *Mammuthus primigenius *and the American mastodon *Mammut americanum*; [see Additional file 1, Fig. S5]). Another uncommon amino acid occurs in most of the *Elephantidae *group at position 266, but not in the American mastodon, suggesting that this mutation originated less than 24 million years ago, after the divergence of the American mastodon from the other four proboscidean species analyzed [36] [see Additional file 1, Fig. S5]). At the N-terminal site 16, adjacent to the Q_i _pocket, there is a positively charged residue (lysine) that will affect the binding of ligands at this site (Figure 9). Residues 260 and 266 are located in loop *ef*, which contributes to the formation of the ISP binding crater. Changes in shape complementarity between the ISP and CytB have been shown to be important for the control mechanism of ISP conformational change, an important feature of cytochrome *bc*_1 _mechanism [37]. The aspartate on position 260, a conserved asparagine spot (Figure 10), may affect both the interaction with the ISP (protein recognition event) and other electron transfer events, as it adds an extra negative charge to this interface (Figure 9). Also on loop *ef*, the shift from the conserved proline on site 266 to a leucine will alter the rigidity of the secondary structure, with implications in the protein-protein interface contacts (Figure 9).

**Figure 11:**
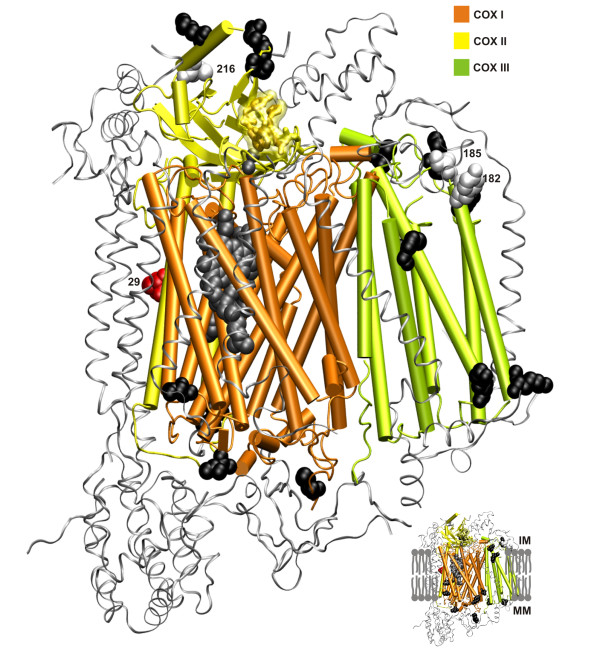
**Three-dimensional representation of variable amino acid in mammalian cytochrome *c *oxidase subunits I, II and III**. Sites within COX subunits showing a high number of amino acid properties positive selected [see Additional file 1, Fig. S4] mapped into the bovine structure (pdb code: 1V54 [67]). Side-chains with over 5 amino acid properties positive selected are presented as black spheres. Sites showing particular mutational trends are shown in white (see text for further details). Mutations in sites shown in red have been related to exercise intolerance in humans (see Table 1). Prosthetic groups are presented as grey spheres. The side chains of the hydrophobic loop in COXII that is involved in cytochrome *c *docking and electron transfer are depicted as sticks together with their van der Waals surface. The nuclear encoded subunits of Complex IV are shown as in silver. MM: mitochondria matrix; IM: intermembrane space.

The cetaceans have an alanine in position 266 (Figure 10), which will have a similar consequence to that referred above. The biochemistry involved in aquatic mammals is still a matter of debate, but it has been found that mitochondria of seals can survive ischemia for much longer than terrestrial mammals [38], suggesting changes in function or regulation. The humpback whale has another interesting mutation. An arginine in site 110 (Figure 10) changes the local net charge, and being located just outside the coordination sphere of heme b_H_, such a difference can interfere with the electron transfer events (Figure 9). Such alteration is also seen in two of the Chiroptera species, the Ryukyu flying fox and the New Zealand long-tailed bat (Figure 11). Curiously, the sloth, one of the mammals showing a lower metabolic rate than expected given its body size [14], has a positively charged residue on site 111 (a lysine instead of the highly conserved glutamate), a yet more radical change in net electric charge at this location (Figure 10).

Another region that shows radical amino acid variation is helix cd2, close to the hinge region of ISP (Figure 9). Mutations in the hinge region were shown to have drastic consequences in the catalytic activity of ISP, by hindering the conformational changes that are required for cytochrome *bc*_1 _function [39]. Two species have a proline residue on site 158: the greater cane rat, a rodent with spiny fur on the back, and the pangolin, the scaly-anteater, which has a low metabolic rate because of the combination of an invertebrate diet and a large body size [14]. Sites 16, 159, 162 and 263 show an elevated number of amino acid changes (Figure 10), suggestive of adaptive relevance in many mammalian species. Site 277 presents a highly conserved alanine residue. It is placed in the middle of helix F1, within the Q_0 _pocket (Figure 9). Two peculiar species (dugong and alpaca) with distinct metabolic requirements show very radical amino acid changes at this site. The dugong is an aquatic mammal that is more closely related to elephants than to other marine mammals [40]. It is sometimes referred to as a sea cow because of its strict sea-grass diet, combining several interesting features from the metabolic point of view: a large body size, a low energy diet and aquatic environment adaptation. It has an arginine residue in position 277, which will not only cause extra steric hindrance because of the size of the side chain relative to alanine (in Figure 9 the van der Waals surface of an arginine side chain is presented; it clearly overlaps the stigmatellin binding position), but will also change the binding mode of the ligand, as it is positively charged. Finally, an important change was detected in the alpaca, a domesticated breed of South American camel-like ungulates that lives at an altitude of 3500 to 5000 meters above sea-level presenting metabolic adaptations to the low O_2 _environment [41]. This species has a proline at site 277, which will drastically alter the local secondary structure, disrupting the alpha helix and therefore changing the shape of the Q_0 _pocket. Such a mutation is not present in the closest relatives of the alpaca [see Additional file 1, Fig. S5]. Curiously, the Old World members of the Camelidae family (the dromedary *Camelus dromedarius *and the bactrian camel *C. bactrianus*) have an aspartate in position 16, instead of the asparagine exhibited by the four species that inhabit South America (alpaca *Lama pacos*, guanaco *L. guanicoe*, llama *L. glama*, and vicuna *Vicugna vicugna*) [see Additional file 1, Fig. S5]. Several mutations in human CytB have been related to exercise intolerance [12] (Table 1; Figure 9), all of which have similar chemical effects including a change in the net charge around the heme groups and in the binding pockets and disruption of local secondary structures close to the substrate binding areas.

### Cytochrome c Oxidase

Complex IV is the terminal electron transfer chain complex that catalyses the electron oxidations of four consecutive reduced cytochrome *c *molecules and the concomitant reduction of one O_2 _molecule to water [42]. COXI and II subunits are directly involved in the electron transfer and proton translocation processes, while the other 11 subunits are thought to have regulatory roles. Results obtained with TreeSAAP were mapped on an available 3D structure for COX (Figure 11). COXI and II are the most conserved of all the 12 subunits analyzed (Figure 3; Figure 4; [see Additional file 1, Fig. S4]). Recent studies on taxa that had clearly experienced significant changes in their metabolic needs detected positive selection acting in COXI and COXII, namely in carnivorous plants [7] and in high-performance fish [6]. As demonstrated in high-performance fish COXII subunits, the highly variable sites in mammals are located at the interface between mitochondrial and nuclear-encoded subunits (Table 3), suggesting either an unknown biological role or the occurrence of compensatory mutations in the nuclear subunits (co-evolution). The known functional spots in the complex were found to be very conserved, namely the portion of COXII directly involved in the electron transfer from cytochrome *c *to the binuclear Cu_A _center (hydrophobic loop consisting of His102-Tyr105 depicted in Figure 11), the negative patch that surrounds it (Asp119, Glu132, Asp139, Glu157, Asp158) [43], the metal binding sites, and the proposed proton/O2/water pathways [44-46]. COXIII had more variable sites, which is expected due to the fact that it has no associated redox cofactors. Also, COXIII has been shown to be unnecessary in some bacterial organisms [45]. Some other interesting changes in COX included the K216E mutation (charge inversion) in COXII observed only in cetaceans, and Y182H in COXIII only seen in the hippopotamus and the dugong (Figure 11). Site 185 in COXIII shows a big variation in amino acid type across all species. Finally, the mutation M29K in COXII has been shown to cause exercise intolerance in humans (Table 1; Figure 11).

**Table 3:** Variable amino acid sites located in COXI/II/III. These sites located on the mtDNA encoded subunits of COX (subunits I, II and III) show significant amino acid properties variation and are in contact with the nuclear encoded subunits of Complex IV (subunits IV, VB, VIA, VIB, VIIA, VIIB, VIIC and VIII).

**COX**	**I**	**II**	**III**
IV	483		

**VB**	**510**		**155, 230**

VIA			38, 122, 155, 182, 185, 230

**VIB**		**92**	

VIIA	4		41

**VIIB**	**406**		

VIIC	4, 502		

**VIII**	**406, 483**		

### ATP synthase

**Figure 12:**
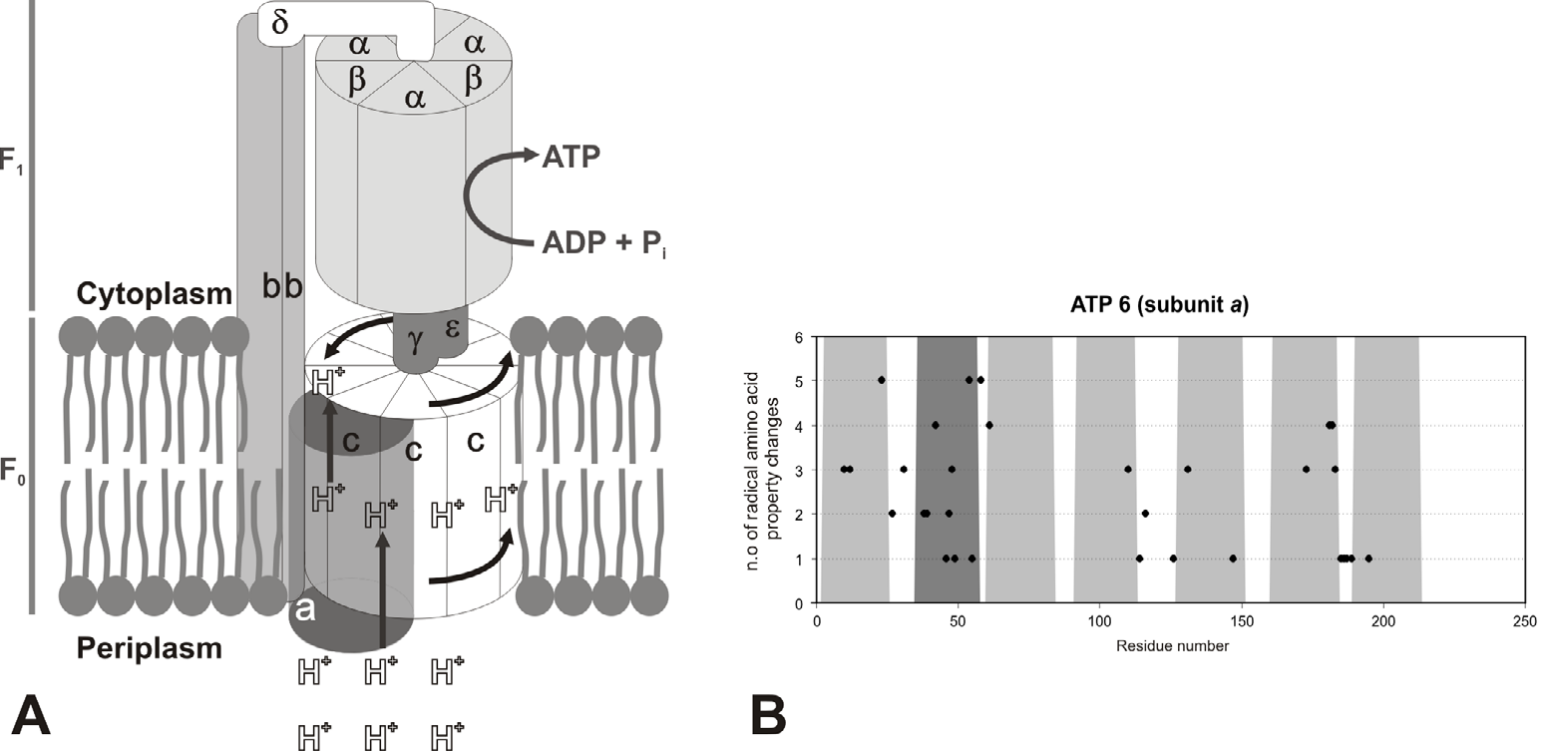
**Rotary model for *E. coli *F_1_F_0 _ATPase and variation in the mammalian ATP6 subunit**. A) Rotary model for *E. coli *F_1_F_0 _ATPase (see text for details); B) Topological assignment of the sites that present a high number of strong positively selected amino acid properties under positive-destabilizing selection in ATP6 (corresponds to the *a *subunit in *E. coli*). The transmembrane domains location is shown in grey (for details see Material and Methods section). The dark grey domain was only predicted by one of the three methods used.

The proton-gradient that results from H^+ ^pumping into the intermembrane space, is used by ATPase to synthesize ATP. The proton channel is located in the membrane sector (F_0_) which is connected to the catalytic component (F_1_), located on the matrix side of the membrane (Figure 12A). The latter, when separated from the membrane, behaves as a soluble ATPase. Large cooperative conformational changes occur in order to couple the passage of protons through the membrane arm and the production of ATP [47,48]. In the proposed mechanism for *E. coli *ATPase, protons that have accumulated in the periplasm enter the assembly via subunit *a *(corresponding to ATP6 in yeast [49]). One proton binds between two *c *subunits (corresponding to ATP9 subunits in yeast [49]). In order for the proton to reach the exit channel, the *c *subunits (in a total of 10 in *E. coli*), that are arranged as a cylinder, have to rotate, releasing the proton after 10 steps of proton binding. This rotation movement involves the γ and ε subunits, that remain fixed to the top of one set of *c *subunits. The rotation of γ within the α/β subunits induces conformational changes that release ATP from the alternating catalytic cycles. The ε subunit (homologous to mitochondrial subunit δ and IF_1 _regulatory protein) is responsible for determining whether complex V acts as a synthase or catalyses the reverse reaction (pumping protons from the cytoplasm/matrix to the periplasm/intermembrane space) at the expenses of ATP hydrolysis. Subunits *b *and δ (equivalent to mitochondrial OSCP) keep the α/β subunits in a fixed position. The conservation of ATP6 reflects its key role in the coupling of the proton flow with the rotation of the *c *subunits: as for the ND complex, the sites with higher variation are located only in the predicted loop regions (Figure 12B).

The ATP8 gene encodes a core subunit of the F_0 _component of ATPase. In its absence, the ATPase in yeast contains no ATP6 subunit, which suggests an important role in the assembly of F_0 _[50]. Nevertheless, ATP8 subunit has some highly variable sites (Figure 12B), presenting the higher average of radically changing amino acid properties per residue, suggesting some variation of its regulatory role across species.

### Oxidative phosphorylation vs metabolic rates

**Figure 13:**
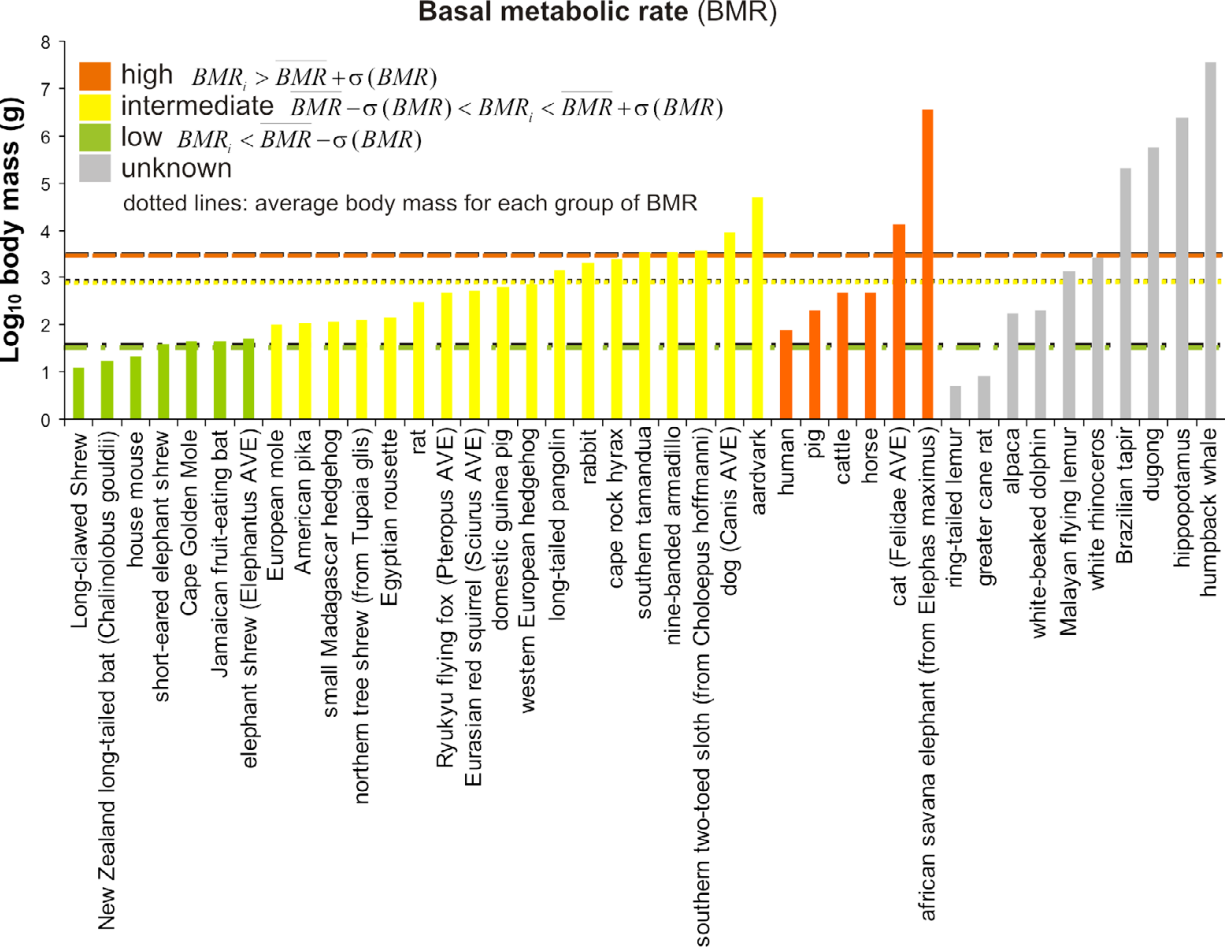
**Basal metabolic rate *vs *log_10 _(body mass)**. The basal metabolic rate (BMR) is presented in three categories (low, intermediate and high). When the BMR value was not available for the species in study, either that of a close relative (indicated in parenthesis) or the average values of several closely related species (AVE) were used (together with the corresponding body mass value). The increase in BMR with the body mass can be easily observed by comparing the average values for each category (dotted lines).

Some mutations in mitochondrial genes are responsible for severe phenotypic effects related to metabolic capacity, such as exercise intolerance in humans [11] (see Table 1). However, the scaling of metabolic rates in relation to the ATP yield through oxidative phosphorylation is not straightforward, as the total metabolic rate is influenced by multiple characteristics varying across species. The scaling of metabolic rates by body size has been discussed for more than a century [13] and remains controversial [13,51-54], although generally metabolic rate increases as body mass increases (see this trend in our dataset: Figure 13 and Table 2). However, there are numerous exceptions such as for large tropical ant and termite predators (e.g. the sloth, the aardvark, some pangolins, tamanduas and armadillos), for which basal rate of metabolism decreases when their size increases [14]. Furthermore, variation in size is accompanied by changes in the metabolic rates inherent to cells (in vitro studies showed that these decline with increasing body mass; [55]). Some of the approaches to scale the whole body basal metabolic rate take into account the scaling of individual organ masses and metabolic rates [56]. However, variation can occur in the activity of oxidative enzymes, such as in the case of acclimatization to altitude by lamas and alpacas [41].

The variation between the metabolic rates in different species is a consequence of multiple factors, including the need to maintain body temperature, the number of mitochondria and the volume densities and/or cristae surface, and the fact that relative organ mass and organ metabolite rate varies interspecifically. For example, in reptiles, the lower metabolic rates, compared to mammals, are due to a combination of smaller internal organs, lower mitochondrial volume and cristae surface densities [55].

The scaling of metabolic rates is thus an intricate issue, and even recent multiple-cause models [52] are flawed [57]. Adding to the complexity of interspecies metabolic rates analysis is the random accumulation of variation in the coding sequences of proteins directly involved in energy production and differential selective pressures that arise as mutations affecting mitochondrial ATP production.

## Conclusion

We present a mammalian phylogeny based on variation in protein-coding mtDNA genes among 41 representative species. Sequence analyses were complemented with functional analyses to assess the potential importance of mutations leading to radical changes in the physicochemical properties of the amino acids. Most of the mtDNA protein-coding genes were extremely conserved, reflecting their vital role in oxidative phosphorylation. However, much of the observed variation had plausible adaptive significance.

The ND2, ND4, and ND5 complex I genes showed higher than average adaptive variation, with all of the variable sites located in the assessed loop regions of these putative protons pumps (3D structural data are needed to further confirm these interpretations and to measure the functional implications).

The available high resolution 3D structure of CytB facilitated interpretation of the functional implications of mutations occurring at portions of the protein which resulted in extreme amino acid properties variation in species with peculiar metabolic requirements (such as adaptation to low energy diet *vs *large body size, namely in elephant, dugong, sloth, and pangolin; and adaptation to extreme O_2 _requirements, i.e. diving in cetaceans, flying in bats, and high altitudes resistance in alpacas). The adaptive variation in COX was restricted mostly to the interface between mitochondrial and nuclear-encoded subunits, suggesting either co-evolution or some influence in the regulatory role of the latter. Among the ATPase subunits, ATP8 which has an important role in the assembly of F_0_, showed the highest amount of adaptive variation in this analysis. ATP6, which has an essential role in the ATPase rotor performance, showed a high adaptive variation in predicted loop areas. Interpretation of possible functional roles of these changes is limited, however, by the lack of experimental and structural data for these genes.

Our study provides insight into the adaptive evolution of the mtDNA genome in mammals, which may have facilitated the successful radiation and diversification of mammalian species into different environments and habits. The evidence of positive selection acting in important functional regions of the various mammalian mtDNA proteins provides the framework for future experimental characterization of the impact of specific mutations in the function, physiology, and interactions of the mtDNA encoded proteins involved in the oxidative phosphorylation.

## Methods

### Phylogenetic analyses

A mammalian mitogenomic phylogeny was constructed using 12 of the 13 protein-coding genes of the mtDNA genome of 41 species representative of all mammalian orders (Table 2). The ND6 gene was excluded because it is encoded by the light-strand which has a significantly different base composition from the heavy-chain [58]. Gaps and ambiguous sites adjacent to gaps were removed, resulting in a total alignment of 10,587 nucleotides (3,529 amino acids). The third codon position was excluded from the phylogenetic analysis (7,058 nucleotides were used) because of observed nucleotide saturation [see Additional file 1, Fig. S1].

Bayesian inference methods with Markov chain Monte Carlo (MCMC) sampling were used in MrBayes [25,26] to assess phylogenetic relationships among the species. We used a General-Time-Reversible substitution model [59] with the invariant site plus gamma options (five categories) after determining the optimal model of sequence substitution with MrModeltest 2.2 [60]. One cold and four incrementally heated chains were run for 2,000,000 generations with chains I = 2, 3, 4, and 5 incrementally heated with heat being 1/(1+ [i-1]T) and T = 0.2. Trees were sampled every 100 generations from the last 1,000,000 generated (well after the chain reached stationarity) and 10,000 trees were used for inferring Bayesian posterior probability. The burn-in fraction performance was evaluated using the program Tracer v1.4 . Bayesian methods have been successfully applied to estimation of the tree topology of placental animals using both mitochondrial and nuclear data [27]. A maximum likelihood phylogenetic tree was constructed in PAUP 4.0b10 [61] after determining the optimal model of sequence substitution (TVM+I+G) with Modeltest 3.04 [62].

### Adaptive evolution analyses

Selection in protein-coding genes is generally assessed by estimating ω, the ratio between nonsynonymous and synonymous substitution rates (*d*_N_/*d*_S_) [63]. However, this statistical approach for detecting molecular adaptation is largely biased against even moderately conservative proteins as it does not allow the possibility that adaptation may come in the form of very few amino acid changes. Thus, significant physicochemical amino acid changes among residues in mitochondrial protein coding genes were identified by the algorithm implemented in TreeSAAP [28], which compares the observed distribution of physicochemical changes inferred from a phylogenetic tree with an expected distribution based on the assumption of completely random amino acid replacement expected under the condition of selective neutrality. The evaluation of the magnitude of property change at nonsynonymous residues and their location on a protein 3D-strcuture may provide important insight into the structural and functional consequences of the substitutions [64]. Eight magnitude categories (1 to 8) represent one-step nucleotide changes in a codon and rank the correspondent variation in a property scale of the coded amino acid. Categories 1 to 3 indicate small variation in the amino acid characteristics while categories 6 to 8 represent the most radical substitutions. By accounting for the property changes across the data set, a set of relative frequencies changes for each category is obtained allowing to test the null hypothesis under the assumption of neutral conditions [65]. The categories for which the observed numbers of amino acid replacements in the data set is significantly different from the null model (z-scores > 1.645; *P *< 0.05) are considered as being potentially affected by selective pressures [65]. Here we focus on amino acid differences that correspond to radical physicochemical variation (positive-destabilizing selection) and are expected to be linked with significant changes in function. TreeSAAP categorizes each amino acid site by positive and negatively destabilizing using 31 properties (henceforth amino acid positions will be referred as sites). To detect strong directional selective pressure, only changes corresponding to categories 7 and 8 (the 2 most radical property changes categories) at the *P *≤ 0.001 level were considered. The total number of changes per site is the sum of those occurring in each branch of the phylogeny. The number of changes in amino acid properties was standardized relatively to the overall size of the protein when comparing different complexes (weight factor = total number of amino acids in the complex/total number of amino acids in ATPase, which is the smallest protein complex).

### Protein structure analyses

The functional relevance of the amino acid mutations was discussed in the context of existing three-dimensional (3D) structures of mtDNA encoded proteins (CytB [PDB:1PPJ] [66]; COX [PDB:1V54] [67]). For those proteins with unknown 3D structures (ND and ATPase), topologies for transmembrane (TM) subunits were predicted using hidden Markov model (HMM) based servers for topology prediction of transmembrane proteins [68-71]. The algorithm used by the program PRODIV-TMHMM [70] has proven to be very reliable at predicting 3D topologies as it incorporates evolutionary information from multiple sequence alignments and assigns amino acid residues to different TM regions according to their properties. However, since even homologous sequences from the same protein family can have inverted topologies [72], some caution is necessary when using topologies predicted by these automated approaches. We have therefore delineated putative TM domains by integrating the results from PRODIV-TMHMM with two other reliable HMM based methods [73], TMHMM [68,69] and HMMTOP [71]. Graphic representations of the 3D structures were created with the program VMD [74].

## List of abbreviations

mtDNA: mitochondrial DNA; ATP: adenosine triphosphate; ADP: adenosine diphosphate; NADH: nicotinamide adenine dinucleotide; ND: NADH dehydrogenase; CytB: cytochrome *b*; COX: cytochrome *c *oxidase complex; ATPase: ATP synthase.

## Authors' contributions

RF performed all phylogenetic, evolutionary and structure-function analyses and drafted the manuscript.

WEJ participated in the drafting and coordination of the study.

SJB participated in the drafting and coordination of the study.

MJR participated in the drafting and coordination of the study.

AA participated in the design, genetic analyses, drafting and coordination of the study.

All authors read and approved the final manuscript.

## Supplementary Material

Additional file 1 Supporting information. Saturation plots (S1), maximum likelihood tree (S2), number of strong positively selected amino acid properties plotted against the branch length (S3), number of strong positively selected amino acid properties across all sites in each mammalian mtDNA-encoded subunit of the respiratory chain (S4) and the sites indicated in Figure 10 in four elephant relatives and 6 alpaca relatives (S5). Click here for file
