# Supplementary material for: The adaptive evolution of the mammalian mitochondrial genome
Source: BMC Genomics. 2008 Mar 4;9:119. doi: 10.1186/1471-2164-9-119 (PMC2375446; doi:10.1186/1471-2164-9-119)
Supplement: Additional file 1 — Supporting information. Saturation plots (S1), maximum likelihood tree (S2), number of strong positively selected amino acid properties plotted against the branch length (S3), number of strong positively selected amino acid properties across all sites in each mammalian mtDNA-encoded subunit of the respiratory chain (S4) and the sites indicated in Figure 10 in four elephant relatives and 6 alpaca relatives (S5). [file 1471-2164-9-119-S1.pdf]

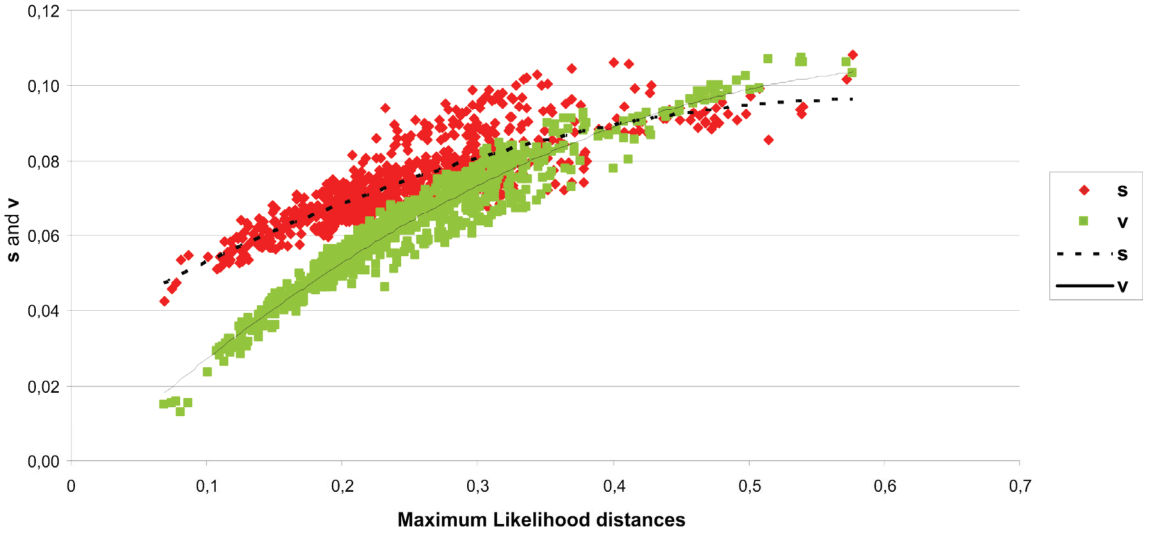

Figure S1.
Saturation plots of transitions (red diamonds; dotted line) and transversions (green squares) calculated through pairwise sequence comparisons. Maximum-likelihood distances calculated using the substitution model that best fitted the data are plotted against the percent number of substitutions all codon positions.

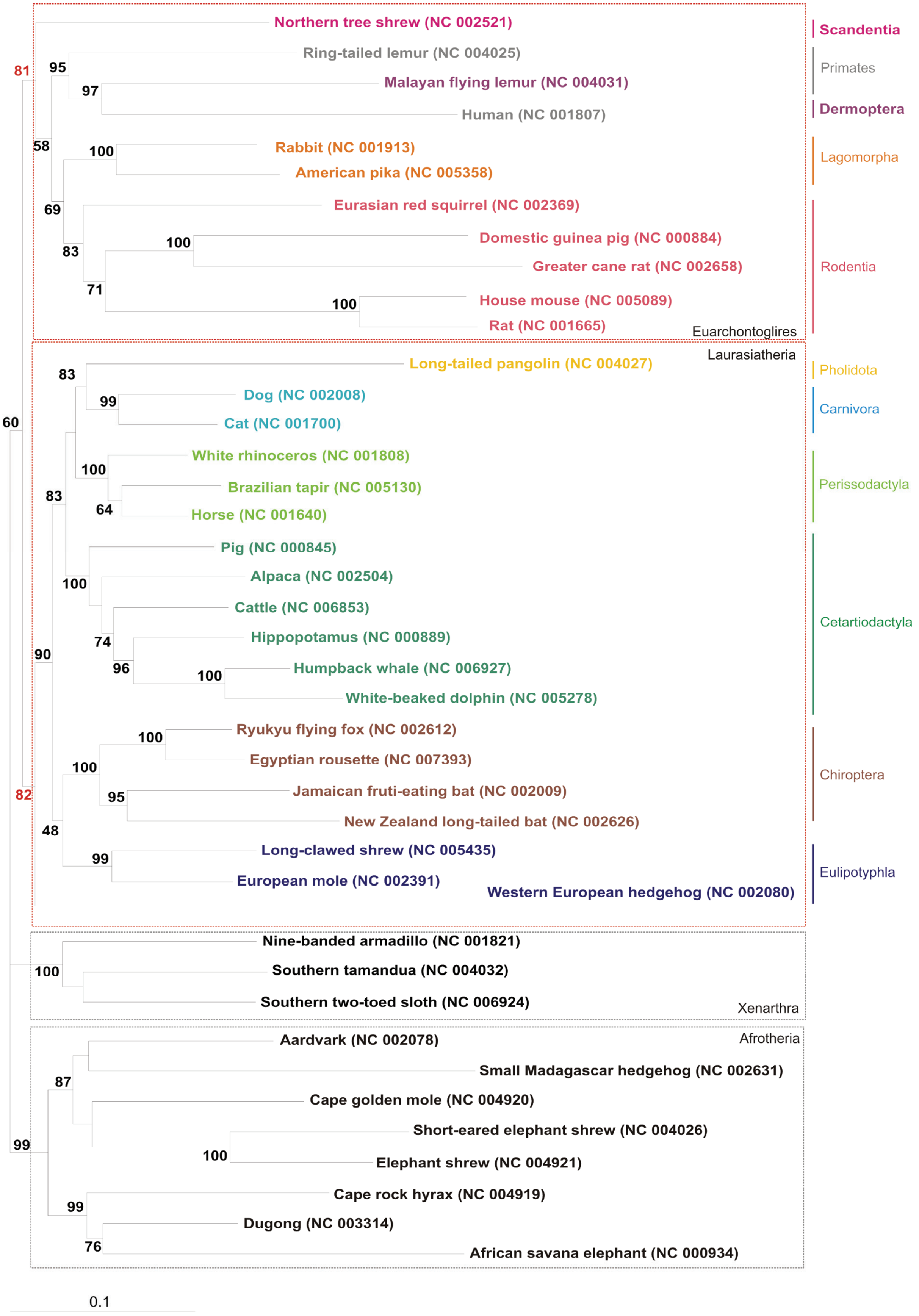

Figure S2.
Maximum likelihood tree obtained using the TVM+I+G model of sequence substitution. Maximum likelihood (ML) bootstrap values were obtained through 1000 pseudoreplicates in PHYML.

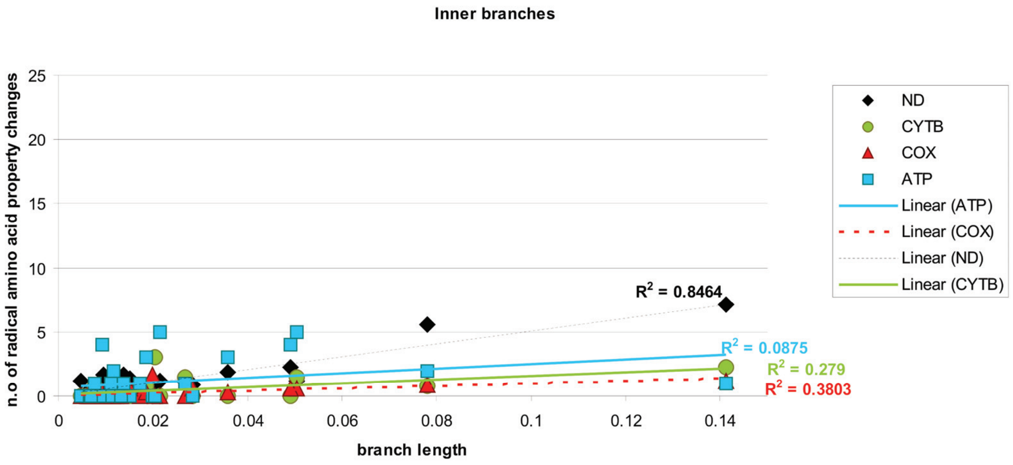

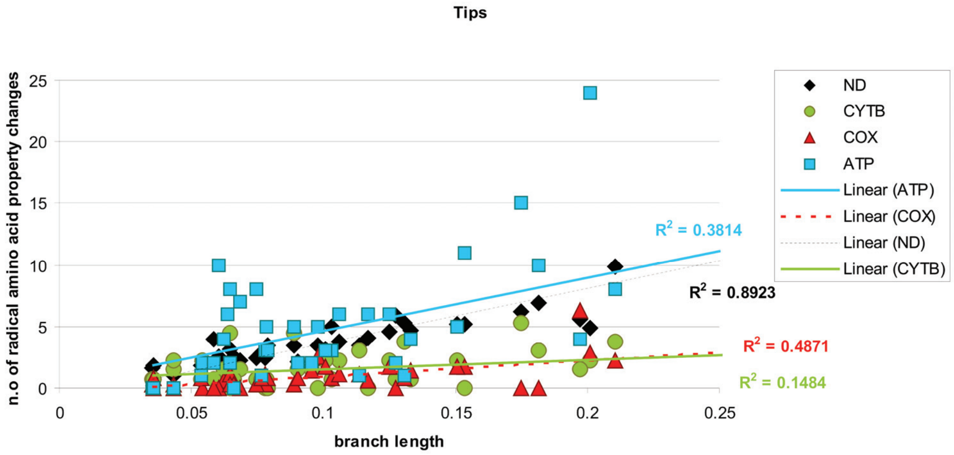

Figure S3.
Number of strong positively selected amino acid properties vs branch length.

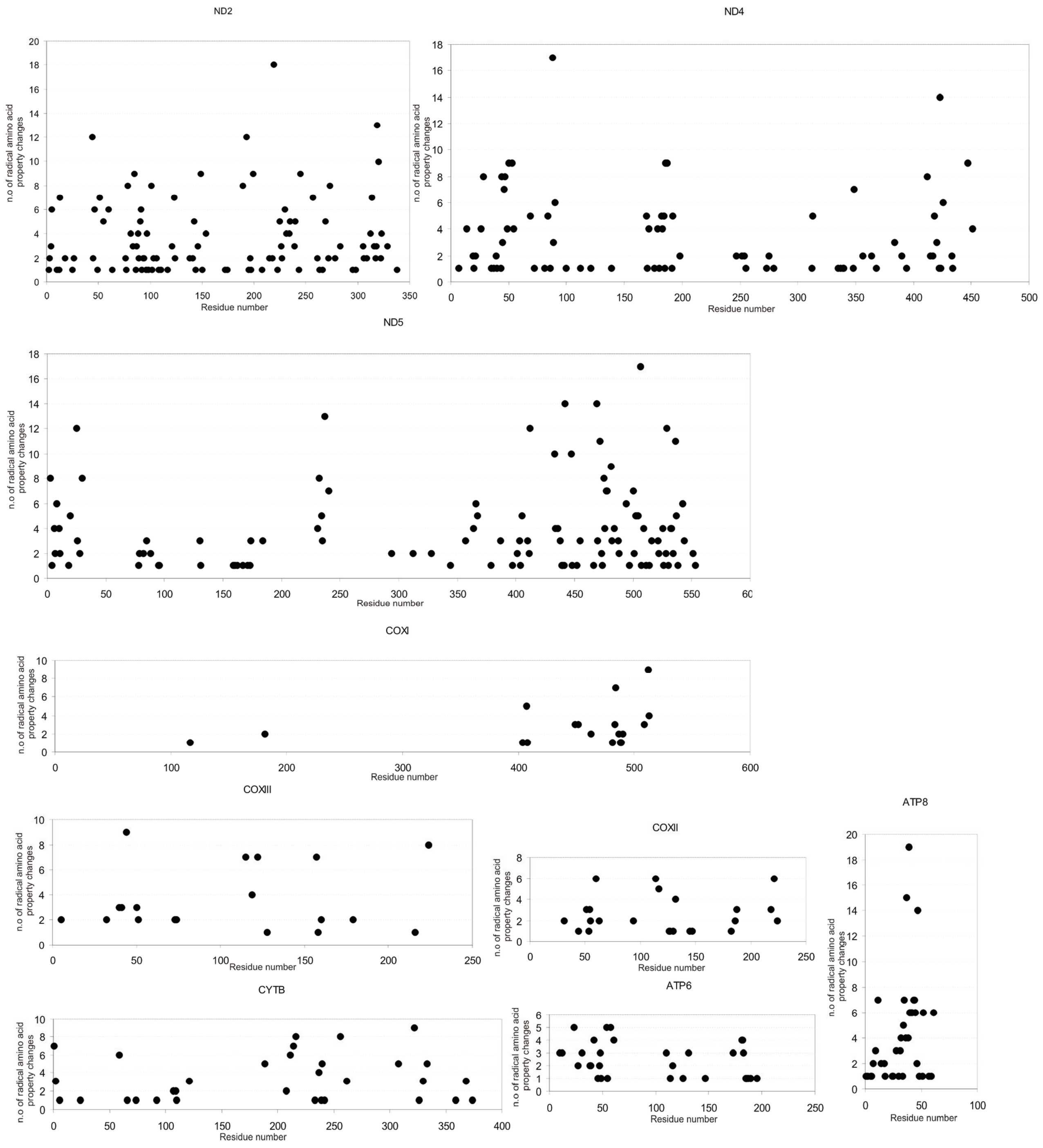

Figure S4.
Number of strong positively selected amino acid properties (y-axis) across all sites (x-axis) in each mammalian mtDNA-encoded subunit of the respiratory chain.

		16 110 111 158 159 162 260 263 266 277
**NC 005129**	*E maximus*	K-SE-TN-E-D-N-L-A
**AJ224821**	*L africana*	K-SE-TN-E-D-N-L-A
**AY741079**	*L cyclotis*	K-SE-MS-E-D-N-L-A
**NC 007596**	*M primigenius*	K-SE-TD-E-D-N-L-A
**NC_009574**	*M americanum*	K-SE-TS-E-D-N-P-A
**U06429**	*L glama*	N-LE-TT-E-N-N-P-A
**Y08812**	*L guanicoe*	N-LE-TT-E-N-N-P-A
**U06430**	*V vicugna*	N-LE-TT-E-N-N-P-A
**NC 002504**	*L pacos*	N-LE-TT-E-N-S-P-P
**U06427**	*C bactrianus*	D-LE-TT-E-N-N-P-A
**U06426**	*C dromedarius*	D-SE-TT-E-N-N-P-A

Figure S5. Sites indicated in Figure 8 in four elephant relatives and 6 alpaca relatives.
